# Clinical picture: multiple sites of ectopic pancreatic tissue

**DOI:** 10.1186/s40064-015-1072-x

**Published:** 2015-06-26

**Authors:** J Straatman, R J Meester, N C T v. Grieken, M J A M Jacobs, P d. Graaf, G Kazemier, M A Cuesta

**Affiliations:** Department of Surgery, VU University Medical Center, Amsterdam, The Netherlands; Department of Pathology, VU University Medical Center, Amsterdam, The Netherlands; Department of Gastroenterology, VU University Medical Center, Amsterdam, The Netherlands; Department of Radiology, VU University Medical Center, Amsterdam, The Netherlands

## Abstract

A case is presented with multiple sites of ectopic pancreatic tissue in the gastro-intestinal tract. The sites were found in the stomach and duodenum, one site of ectopic pancreatic tissue presented with necrotizing pancreatitis. Ectopic pancreatic tissue can be defined as all pancreatic tissue, with no anatomical or vascular continuity with the orthotopic pancreas. The ectopic tissue most likely originates from the spreading of cells, during the allocation of structures derived from the foregut in the embryonic phase. Over ninety percent of ectopic tissue presents in the upper gastrointestinal tract, although other locations have been described. To date this is the first case-report about a patient with multiple localizations of ectopic pancreatic tissue.

## Background

Pancreatic tissue, with no anatomical or vascular continuity with the orthotopic pancreas, is described as aberrant pancreas or ectopic pancreatic tissue. The prevalence of ectopic pancreatic tissue is reported to be around 0.25% in the general population, whereas a prevalence of around 14% is reported in autopsy material (Dolan et al. 1974; Tanaka et al. 1993). The presence of ectopic pancreatic tissue is usually asymptomatic.

Ectopic pancreatic tissue is most often reported in the stomach, duodenum and jejunum, yet many other locations have been reported (Wlaz et al. 2014). Multiple case reports depict different presentations of ectopic pancreatic tissue, describing only singular sites. To our knowledge this is the first case of ectopic pancreatic tissue presenting at multiple sites.

## Case-report

A 61-year-old woman presented to the outpatient clinic of the VU University Medical Center (VUmc), Amsterdam, the Netherlands, with complaints of dysphagia. Computer tomography (CT) scan and magnetic resonance imaging (MRI) of the neck and chest depicted a right dorsolateral para-esophageal mass near the upper esophageal sphincter. Dimensions were 13 mm axially and 35 mm cranio-caudally. The mass was confirmed on endoscopic ultrasound and seemed to be expanding from the muscularis propria. Based on these findings the mass was suspected to be a leiomyoma.

The patient underwent surgical exploration of the neck, searching for suspected leiomyoma of the cervical esophagus. During surgery the tumor could not be identified, even with peri-operative endoscopic ultrasound.

Although no tumor was identified during surgery, the patient presented with progressing complaints of dysphagia. Multiple gastroscopies were performed with dilation of the esophagus, with good effects on the patient’s complaints.

Follow-up endoscopy was performed 1 year later. During the examination a small mass with a central opening was visible in the antrum of the stomach. These findings are consistent with ectopic pancreatic tissue with an accessory pancreatic duct. Biopsies were performed, but quality of the biopsies was too poor to determine the diagnosis. The mass was monitored with bi-annual endoscopy.

**Figure 1:**
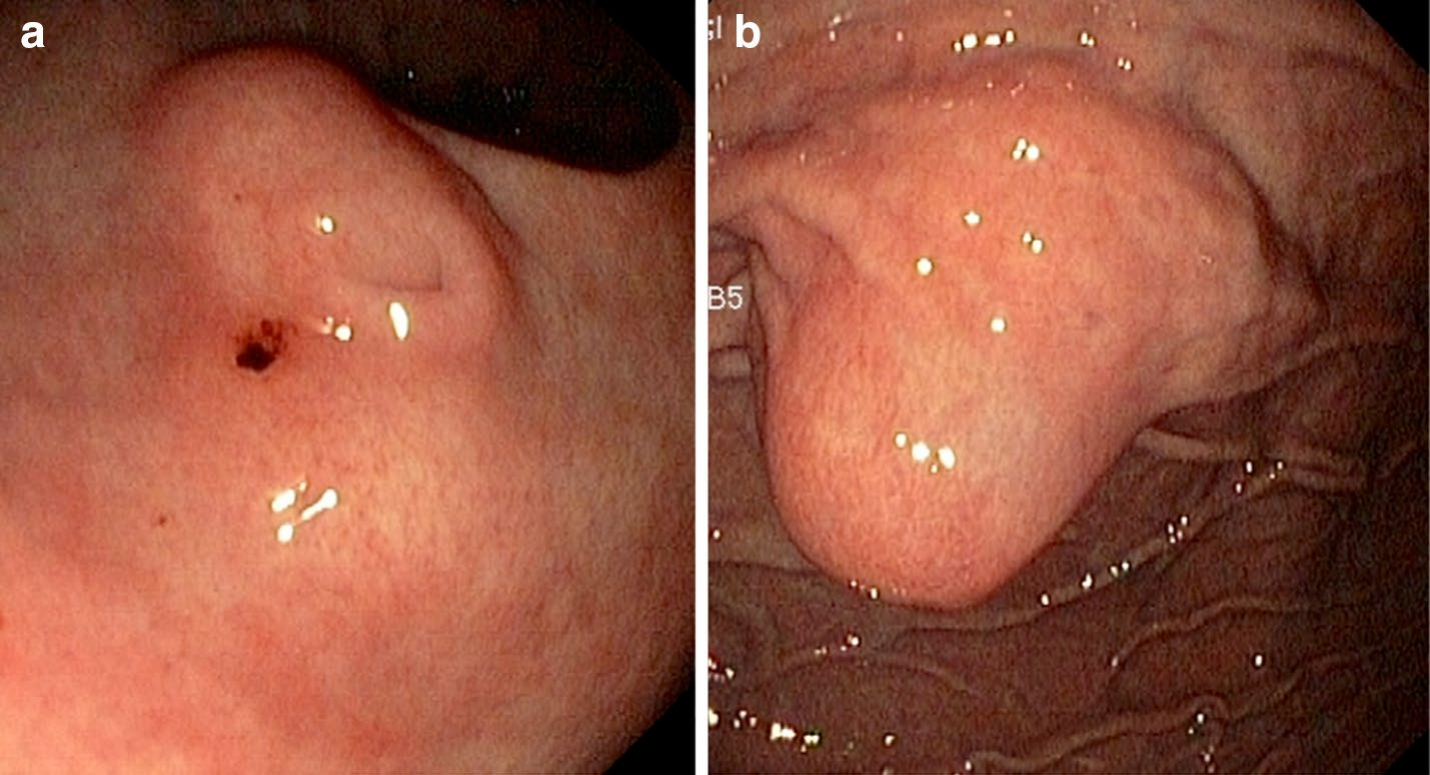
**a** Aberrant pancreatic tissue of the stomach with duct and **b** suspected neuro-endocrine tumour, which was a second site of ectopic pancreatic tissue upon analysis.

Three years later the patient presented with microcytic anemia. Endoscopy was repeated and showed an ulcerated submucosal tumor in the fundus of the stomach. The previously identified mass in the antrum was still present and unchanged in comparison to the site found 3 years ago (Figure 1).

Microscopic analysis depicted normal gastric mucosa in the fundus. In the biopsy from the antrum pancreatic tissue was identified. Additional computer tomography (CT)-scan imaging was performed; showing the tumor in the fundus was centrally calcifying. These findings are consistent with a neuro-endocrine tumor and an indication for resection.

**Figure 2:**
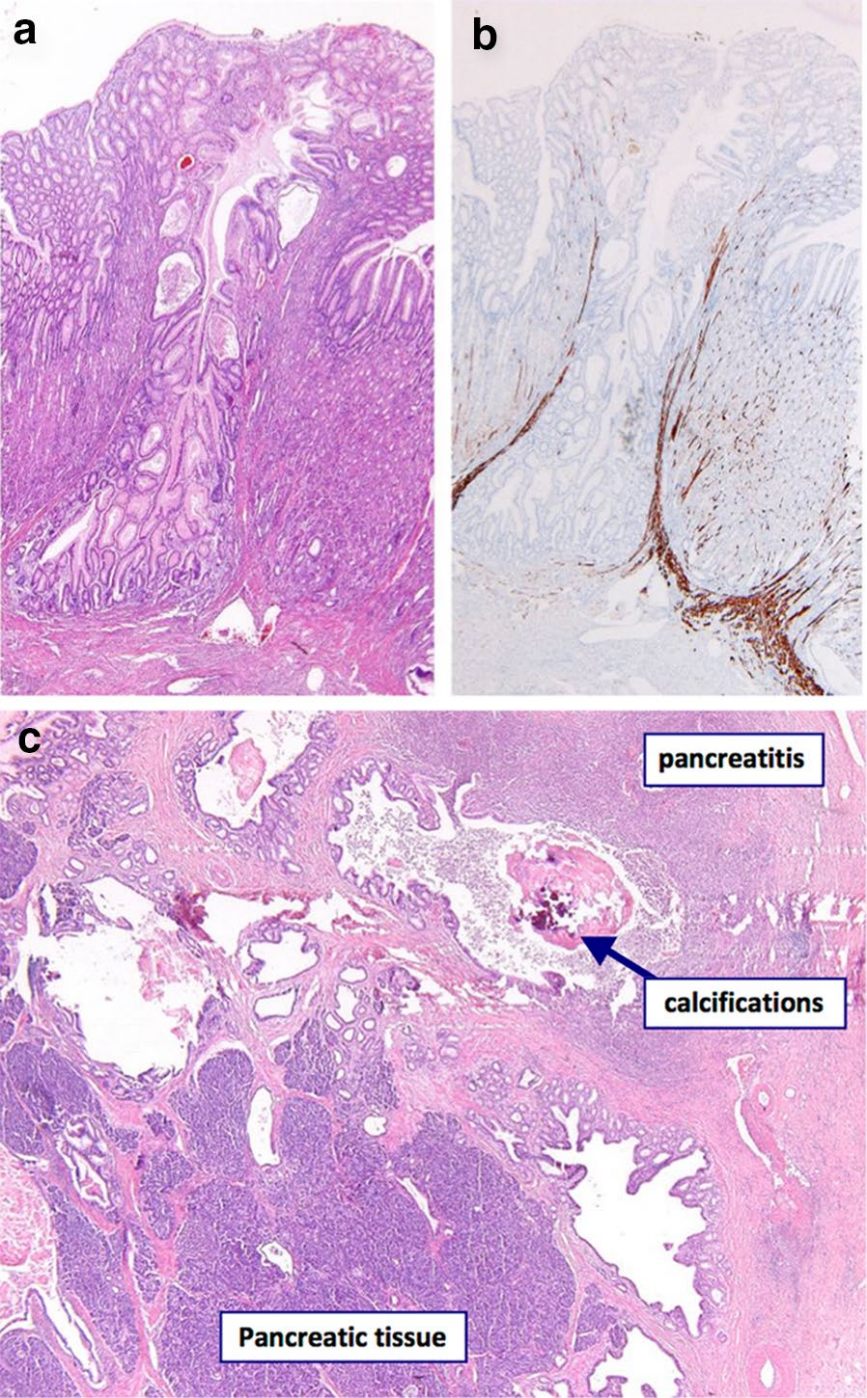
**a**, **b** Histology of the wedge resection specimen, displaying a pancreatic duct in ectopic pancreatic tissue. **c** Histology of the wedge resection specimen, displaying pancreatitis in ectopic pancreatic tissue of the stomach.

A laparoscopic wedge resection of the mass in the fundus was performed. Postoperative recovery was uncomplicated. The pathologist described a mass of ectopic pancreatic tissue with a central duct and acute necrotizing pancreatitis (Figure 2).

Two sited of ectopic pancreatic tissue were now confirmed, one being the aberrant tissue with accessory duct in the antrum of the stomach, the second being the tumor in the fundus with necrotizing pancreatitis.

Following these results, the tumor in the esophagus was studied with MRI under suspicion of a third site of ectopic pancreatic tissue. The MRI image depicted the esophageal mass near the upper esophageal sphincter, compatible with a benign tumor. As the patient had no progression of complaints it was decided that re-exploration of the cervical esophagus was not necessary.

The patient is followed-up bi-annually for assessment of the sites in the esophagus and stomach with endoscopy. The last endoscopy revealed two new sites of ectopic pancreatic tissue, one being in the stomach and another site was observed in the duodenum. As all sites are asymptomatic, resection is not deemed indicated.

## Discussion

The clinical incidence of ectopic pancreatic tissue is rare and believed to be 0.25% (Tanaka et al. 1993). Whereas rates have been described to be as high as 13.7% in autopsy material, indicating a high incidence of asymptomatic ectopic pancreatic tissue (Dolan et al. 1974). Symptomatic patients often present with wide range of symptoms, such as abdominal pain, dysphagia and in rare complicated cases patients present with pancreatitis, pseudocysts, upper gastrointestinal bleeding or even adenocarcinoma of ectopic pancreatic tissue (Osanai et al. 2001; Lee et al. 2014).

The ectopic tissue most likely originates from the embryonic phase, spreading of cells during the allocation of the structures originating out of the foregut (Lai and Tompkins 1986). The exact pathology is unknown but theories support wrong positioning of the pancreatic tissue during rotation of the foregut or wrongly distributed neuroendocrine cells (amine precursor uptake decarboxylase cells) in the embryonic phase as origin of the aberrant pancreas (Zinkiewicz et al. 2003).

As described above, the incidence of ectopic pancreatic tissue, without symptoms, is high. Indicating, ectopic pancreatic tissue does not require resection or other treatment if no symptoms are present (Dolan et al. 1974). The ectopic tissue in the antrum of the presented patient is asymptomatic, it was visualized 3 year prior to the resection of the mass in the fundus upon routine endoscopy due to complaints of dysphagia. Symptoms are usually associated with pancreatitis, stenosis or malignancy.

Previous case-reports describe pancreatitis, intussusception, neuro-endocrine tumors, bleeding and obstruction arising from ectopic pancreatic tissue (Razi 1966; Okasha et al. 2013; Habibi et al. 2014; Monier et al. 2014).

With regard to localization 90% of ectopic pancreatic tissue sites are found in the upper gastrointestinal tract, with 27.5% of cases arising in the stomach (Wlaz et al. 2014). Sites have also been reported in the esophagus, ileum, colon, mesentery and omentum (Mortele et al. 2006). Ectopic pancreatic tissue of the stomach may be hard to distinguish from mesenchymal tumors such as leiomyoma or gastrointestinal stromal tumors (GIST) (Park et al. 2011). Diagnostic studies should differentiate between premalignant sites and innocent ectopic pancreatic tissue. Ectopic pancreatic tissue may present differently on ultrasound, CT and MRI. Definitive diagnosis may be obtained with histology following fine-needle aspiration or resection (Attwell et al. 2015).

## Conclusion

Previous case-reports describe singular sites of ectopic pancreatic tissue, to our knowledge this is the first reported case of a patient presenting with multiple sites of symptomatic ectopic pancreatic tissue (Carvalho et al. 2014; Filip et al. 2014; Macedo et al. 2014).
